# Editorial: The neurology and neurobiology of neonatal abstinence syndrome

**DOI:** 10.3389/fped.2023.1201352

**Published:** 2023-05-02

**Authors:** Kelly J. Clemens, Ju-Lee Oei, Lauren L. Jantzie

**Affiliations:** ^1^ School of Psychology, University of New South Wales, Kensington, NSW, Australia; ^2^ School of Women’s and Children’s Health, University of New South Wales, Kensington, NSW, Australia; ^3^ Division of Neonatal-Perinatal Medicine, Department of Pediatrics, Johns Hopkins University School of Medicine, Baltimore, MD, United States

**Keywords:** neonatal brain, phenobarbital, methadone, sex differences, opioid, buprenorphine, gabapentin, foetal

**Editorial on the Research Topic** The neurology and neurobiology of neonatal abstinence syndrome

Neonatal Abstinence Syndrome (NAS) is a global public health crisis (1). For decades, attention was focused on preventing infants from succumbing to undertreated or mistreated withdrawal. However, with improved treatment leading to increased survival of infants with NAS, the long-term impact of prenatal drug exposure particularly from opioids, has become a major international concern (1). An increasing body of evidence, from pre-clinical to clinical and large-scale population studies show that NAS or the exposures leading to NAS, has enduring impact on the child, particularly of the developing nervous system. Children are at an increased risk of cognitive difficulties, mental health issues, and other physical problems linked to poor adult outcomes, including future use of addictive substances (2, 3). The challenge for the scientific community is to understand this relationship and to develop strategies to prevent and treat the long-term neurological problems associated with NAS.

In the first paper of this series, Kushnir et al. focus on the immediate outcomes for infants with NAS, offering a retrospective analysis of infant hospital stays and duration of withdrawal treatment, depending on the combined or concurrent use of the barbiturate phenobarbital. Administered as a sedative and anti-seizure medication, phenobarbital is often used in combination with postnatal opioid treatments (e.g., methadone) or as a second-line treatment if the opioid-based approach is ineffective (i.e., rescue treatment). Here reveal that despite the perceived benefits of phenobarbital as a sedative, its use—especially as a rescue treatment—is linked to longer hospital stays and ongoing treatment, irrespective of whether the mother was an opioid or multi-drug user. Such findings indicate that careful consideration of the longer-term outcomes for the infant is necessary when deciding on the use of second-line treatments.

In the second paper, Yen and Maron provide an overview of this broader impact of prenatal opioid exposure, highlighting the emerging evidence that prenatal drug exposure negatively impacts metabolism and energy requirements, including dysregulation of brain circuitry important for food intake and feeding behaviours. Furthermore, new data from this group showed the potential for sex-specific difference in the expression of genes linked to dysfunctional reward processing. Together this draws attention the broader, long-lasting impact of prenatal opioid exposure, implicating feeding, growth, metabolism and cardiovascular dysfunction, with a particular emphasis on the role of inflammation in these processes.

The impact of these changes is most readily detected in the brain where prenatal drug exposure has been linked to a range of poor neurological outcomes that can influence development in childhood. Using resting-state functional MRI (fMRI) in 3-month-old infants, Radhakrishnan et al. demonstrate changes in global brain network activity in infants with prenatal opioid exposure. Notably, the extent of these changes correlated with maternal psychological factors such as depression anxiety or post-traumatic stress disorder (PTSD), linking infant brain development with maternal mental health.

This emphasis on reward circuitry is extended into the study of Boggess et al. Pregnant mice were treated with either buprenorphine or gabapentin. Post-mortem microscopy and analysis of the offspring brain at weaning revealed long-lasting alterations in excitatory and inhibitory synaptic populations in brain regions important for reward processing. These findings suggest prenatal drug exposure disrupts plasticity in the developing brain, and they further link this to altered reward processing and a susceptibility towards addiction in adulthood.

Overall, these studies support prenatal drug exposure and NAS for producing both acute and long-term syndromes characterised by changes to the central nervous system and systemic physiology. This is a particularly challenging area as outcomes in vulnerable children are often confounded by many lifestyle issues. The authors of the above studies highlight multiple factors that influence the research area, including recruiting and retaining pregnant people into studies and long-term assessments. As a consequence, studies are often informative, yet underpowered, with longitudinal data difficult to obtain. Humans are notorious poly-drug users, with many studies of prenatal drug exposure unable to control for exposure to legal substances such as nicotine or alcohol, each of which alone negatively impacts on fetal development. Other significant variables include maternal mental health, socioeconomic status, education, and employment. Preclinical studies such as that outlined by Boggess et al. offer increased control over these variables, enabling a framework through which a more sophisticated understanding of the neurobiological changes that occur as a consequence of prenatal drug exposure can be developed, and a platform against which pharmacological treatments can be assessed. However, in the absence of real-world data, interpretation is restricted.

Taking these limitations into account, the future of research in this area requires large-scale longitudinal tracking of children exposed to drugs of abuse *in utero* and assessments throughout the lifespan. Only then will a clear understanding of the trajectory of prenatal drug use be gained, and a tailored approach to early interventions will be possible.
